# Carbon dioxide adsorption in open nanospaces formed by overlap of saponite clay nanosheets

**DOI:** 10.1038/s42004-020-00346-5

**Published:** 2020-07-21

**Authors:** Kiminori Sato, Michael Hunger

**Affiliations:** 1 Department of Environmental Sciences, Tokyo Gakugei University, Koganei, Tokyo 184-8501 Japan; 2 Institute of Chemical Technology, University of Stuttgart, 70550 Stuttgart, Germany

**Keywords:** Sustainability, Two-dimensional materials, Solid-state chemistry, Structural properties

## Abstract

Nanoscale open spaces formed by partial overlap of two-dimensional nanosheets in clays, abundantly and ubiquitously available, possess reactive molecular sites such as nanosheet edges in their interior. Here, the capture and storage of CO_2_ molecules in open spaces within saponite clay are explored by solid-state nuclear magnetic resonance coupled with open space analysis using positronium. CO_2_ physisorption occurs on the nanosheet surfaces inside the open spaces under ambient conditions. Thereby, CO_2_ molecules are activated by picking off weakly-bound oxygen from octahedral sites at the nanosheet edges and carbonate species are stabilized on the nanosheet surfaces. This instantaneous mineral carbonation and CO_2_ physisorption occurs in the absence of an energy-consumption process or chemical solution enhancement. This finding is of potential significance for CO_2_ capture and storage and presents an approach of environmentally friendly recycling of low contaminated soil in Fukushima.

## Abstract (web summary)

Sequestration of CO_2_ in geological reservoirs holds great potential, but ex situ mineral carbonation generally requires harsh, energy-intense conditions. Here the authors study CO_2_ physisorption at open nanospaces within overlapping nanosheets in saponite clay under ambient conditions.

## Introduction

Owing to the significant climate change caused by the steady increase of CO_2_ in the atmosphere along with industrial activity^1^, the technology of CO_2_ capture and storage with respect to less-energy intensiveness, cost effectiveness, and environmental friendliness has been long-awaited. In power plants, electricity is generated by burning fossil fuels such as coal and natural gas, which could be a large point source yielding ~26% of global CO_2_ emission through the combustion process^2^. An approach to reduce CO_2_ emission is the efficient capture of CO_2_ and its storage in a stable manner before release to the atmosphere. There are three pathways for CO_2_ capture: pre-combustion capture, oxyfuel combustion, and post-combustion capture^3^. Pre-combustion capture is the decarbonation process before combustion, in which primary fuels are converted into a mixture of hydrogen and CO_2_ using gasification or reforming. In oxyfuel combustion, fuels are burned with an oxygen-enriched gas mixture instead of air, resulting in flue gases that mainly comprise CO_2_ and H_2_O. Post-combustion capture removes diluted CO_2_ from the flue gases produced by the combustion of fuel in the air, which could be applied to most conventional power plants owing to its adaptable operation.

Post-combustion technology for CO_2_ capture is based on the fundamental process of mainly chemical and physical absorption with absorbents of high CO_2_ solubility, and physical adsorption employing solid sorbents^3^. Chemical absorption is the CO_2_ capture process involving the reaction of CO_2_ with chemical solvents in aqueous solution, whereas CO_2_ is absorbed into solvents by applying pressure to promote physical absorption. In the standard environment of power plants, flue gases contain 12–14 vol.% of CO_2_ and are emitted under atmospheric conditions, therefore, they require treatment at elevated pressure to promote CO_2_ removal^4^. Physical adsorption utilizes the physisorption of CO_2_ molecules onto the surface of an adsorbent via the quadrupole interaction in addition to the size effect, thus requiring microporous materials with a high specific surface area^5^. At present, physical adsorption of CO_2_ is still in the early research stage together with the subject as to the thermal and chemical stability of the sorbed CO_2_, mechanical strength as well as production cost.

Another important factor of CO_2_ capture and storage is the long-term sequestration of CO_2_, where CO_2_ storage in geological reservoirs has been often considered^6^. In this approach, CO_2_ is injected into a deep geological formation to be physically confined below an impermeable or very low permeability caprock, such as a shale, allowing for a sequence of possible trapping mechanisms^7^. A fraction of injected CO_2_ is fixed as thermodynamically stable mineral carbonates, as e.g., CaCO_3_ or MgCO_3_ in the geological formation via the reaction with alkaline minerals there^8^. The formation of stable carbonates in the deep underground geology, known as in situ mineral carbonation^9^, could be suitable for long-term storage of CO_2_^10,11^. On the contrary, ex situ mineral carbonation is the above-ground process involving the reaction of CO_2_ with alkaline earth metals extracted from basic rock^12^. Ex situ carbonation is generally conducted using acidic solutions at high temperature/pressure to accelerate the reaction between CO_2_ and materials, resulting in an energy-intensive process that generates a number of liquid wastes^12,13^.

**Fig. 1:**
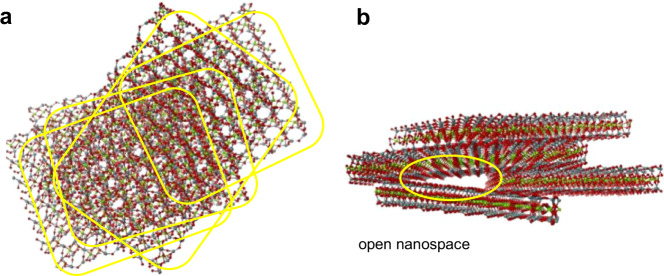
Schematic illustrations of stacked 2D clay nanosheets. **a** Partial overlap viewed from the direction perpendicular to the surface of 2D nanosheets marked by solid squares. **b** Cross-section view. Note that the nanoscale open space is locally formed as marked by a solid circle. Red, gray, and green atoms correspond to oxygen, carbon, and magnesium, respectively.

Saponite, a silicate clay mineral abundantly and ubiquitously available in nature, is structured through stacks of 2D nanosheets with thicknesses of a few nm, which are the minimum structural unit. The 2D nanosheets have a variety of sizes and cannot be perfectly stacked, resulting in partial overlapping as schematically illustrated in Fig. 1a, which has been observed in images of field-emission type scanning electron microscopy^14^. This results in the formation of nanoscale open spaces (see Fig. 1b), which have been identified by positronium (Ps) annihilation spectroscopy together with molecular dynamics simulation as is detailed later^15–17^. Naturally, there exist local molecular sites in the interior of above open spaces such as nanosheet edges that are chemically active owing to the presence of unpaired electrons at ionically bound octahedron^18^. In the present work, CO_2_ adsorption in the open spaces originated from overlapped nanosheets in saponite clay minerals is explored by solid-state nuclear magnetic resonance (NMR) spectroscopy, open space analysis using positronium (Ps), and Fourier transform infrared (FT-IR) spectroscopy coupled with conventional chemical techniques. Besides the Na-type saponite, the Cs type is studied to find out an approach of environment-friendly recycling for low contaminated soil in Fukushima. The emergence mechanism of instantaneous mineral carbonation together with the physisorption of CO_2_ gas molecules, feasible in the absence of energy-consumption process as well as chemical solution enhancement, is highlighted as a future strategy of CO_2_ capture and storage.

## Results

### CO_2_ adsorption on 2D nanosheet surfaces

Chemical analysis by inductively coupled plasma (ICP) spectroscopy indicated that the Cs-type saponite contained ~0.04 mmol/g Cs (see *c*_cesium_ in Table 1). According to a recently developed analytical method using the data of elution test, ^133^Cs magic-angle spinning (MAS) NMR, and radiocesium interception potential, the local molecular structures, as e.g., nanosheet surface, nanosheet edge, and oncoming hexagonal cavity, have been shown to act as Cs adsorption sites^18,19^. In this analysis, 69% of the loaded Cs are found to physisorb on the surface of the 2D nanosheet, which amounts to a concentration of ~0.03 mmol/g. The carbon, hydrogen, and nitrogen (CHN) elemental analysis revealed that the C contents, *c*_carbon_, in the Na- and Cs-type saponite samples after CO_2_ loading are ~0.02 and ~0.18 wt.%, respectively (see Table 1). In light of the fact that the samples are isolated from the air during CO_2_ loading, the C contents detected in the CHN analysis are solely associated with CO_2_. Therefore, CO_2_ molecules are sorbed to the Na- and Cs-type samples at concentrations of ~0.02 and ~0.15 mmol/g, respectively (see *c*_CO2_ in Table 1). The concentration of CO_2_ higher than that of Cs on the nanosheet surface implies the adsorption of several CO_2_ molecules at the Cs cation sites, which will be discussed more in detail later.

**Table 1:** Concentrations of loaded Cs, *c*_cesium_, carbon, *c*_carbon_, loaded total CO_2_, *c*_CO2_, CO_2_ physisorption, *c*_Phys_CO2_, and CO_2_ chemisorption, *c*_Chemi_CO2_.

	*c*_cesium_ (mmol/g)	*c*_carbon_ (wt. %)	*c*_CO2_ (mmol/g)	*c*_Phys_CO2_ (mmol/g)	*c*_Chemi_CO2_ (mmol/g)
Na type	0.00	0.019	0.02	0.014	0.002
Cs type	0.04	0.183	0.15	0.134	0.019

Ps lifetime spectroscopy prior to CO_2_-loading reveals two kinds of open spaces for both the Na- and Cs-type saponite samples. The similar sizes of small and large open spaces with *R*_1_ ~ 3 Å and *R*_2_ ~ 9 Å are obtained for the Na- and Cs-type samples (see Table 2). Our former studies revealed that the above two open spaces commonly observed for both the Na- and Cs-type saponite samples are caused by overlapped nanosheets: the small and large open spaces are the consequence of one- and two-nanosheet insertion into the interlayer spaces^15^. The relative intensity of large open space *I*_2_ for the Cs-type sample is ~25%, much higher than that of the Na-type sample, though the intensities *I*_1_ of small open spaces at 5% are similar to each other. The high intensity *I*_2_ for the Cs-type sample indicating the large amount of 9 Å open space is resultant from insufficient self-assembly toward densification, which originates from interlayer Cs cations with low hydration degree^16,17^.

**Table 2:** Sizes of two kinds open spaces *R*_1_ and *R*_2_ together with their corresponding relative intensities *I*_1_ and *I*_2_ obtained for the Na- and Cs-type saponite samples. The error bars of R1 and R2 are ± 0.06 Å and ± 0.3 Å, respectively.

	*R*_1_ (Å)	*I*_1_ (%)	*R*_2_ (Å)	*I*_2_ (%)
Na type	3.2	6	9.4	9
Cs type	3.3	5	9.1	25

The error bars of *R*_1_ and *R*_2_ are ± 0.06 Å and ± 0.3 Å, respectively.

**Fig. 2:**
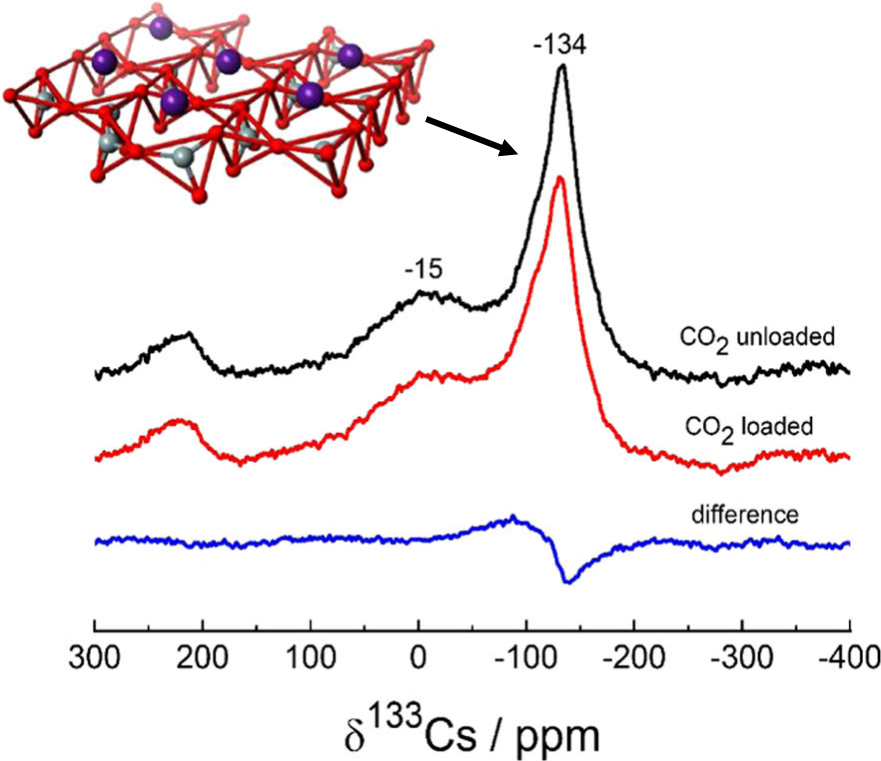
^133^Cs MAS NMR spectroscopy. ^133^Cs MAS NMR spectra obtained for the Cs-type saponite before (black curve) and after (red curve) CO_2_ loading, and their difference spectrum (blue curve). The inset is schematic illustration of Cs^+^ cations (purple) located on the surface of tetrahedral sheet consisting of oxygen (red) and carbon (gray) atoms.

Figure 2 shows the ^133^Cs MAS NMR spectra obtained for the Cs-type saponite before (black curve) and after CO_2_ loading (red curve), and their difference spectrum (blue curve). The dominant signal at ~−134 ppm observed for the sample prior to CO_2_ loading correspond to the Cs^+^ interlayer cations physisorbed on the surfaces of the 2D nanosheets, whereas the additional broad signal at ~−15 ppm originates from Cs_2_O compounds at nanosheet edges^18–20^. The dominant peak arising from the Cs^+^ interlayer cations is slightly shifted and becomes broader upon CO_2_ loading, which can be seen in the difference spectrum as well. This demonstrates that the CO_2_ molecules adsorb at the Cs^+^ cation sites on the nanosheet surfaces at a concentration of ~0.03 mmol/g, as estimated above.

### Physisorption and chemisorption of CO_2_ molecules

**Fig. 3:**
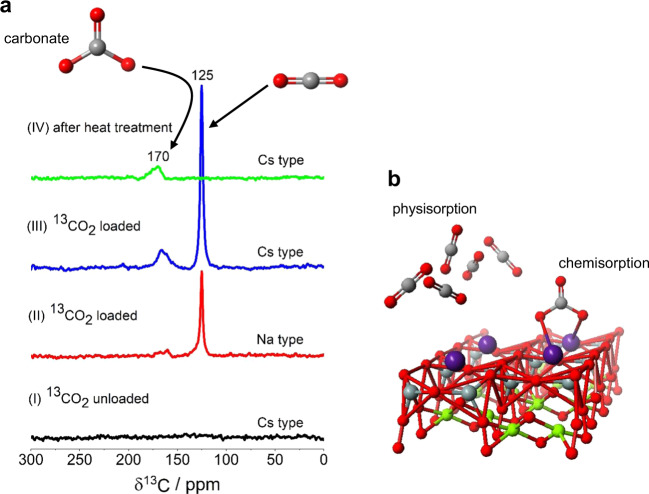
^13^C MAS NMR spectroscopy. **a** ^13^C MAS NMR spectra obtained for (I) Cs-type saponite ^13^CO_2_ unloaded, (II) Na-type saponite ^13^CO_2_ loaded, (III) Cs-type saponite ^13^CO_2_ loaded, and (IV) Cs-type saponite ^13^CO_2_ loaded and then heat treated at 200 °C for 2 h in the N_2_ atmosphere. **b** Schematic illustration of CO_2_ physisorption and optimized structure of Cs_2_CO_3_ as chemisorption on the nanosheet surface. Red, gray, green, and purple atoms correspond to oxygen, carbon, magnesium, and cesium, respectively.

Figure 3a shows the ^13^C MAS NMR spectra obtained for (I) Cs-type saponite ^13^C-enriched CO_2_ (^13^CO_2_) unloaded, (II) Na-type saponite ^13^CO_2_ loaded, (III) Cs-type saponite ^13^CO_2_ loaded, and (IV) Cs-type saponite ^13^CO_2_ loaded and subsequently heat treated at 200 °C for 2 h in the N_2_ atmosphere. Before loading ^13^CO_2_, no signal is observed in the ^13^C MAS NMR spectrum of the unloaded Cs-type saponite. Similarly to that, no signal appears in the NMR spectrum of unloaded Na-type saponite (data not shown here). Upon ^13^CO_2_ loading, an intense peak and broad hump arising from ^13^CO_2_ adsorption appeared at around the chemical shifts of 125 and 170 ppm (see (II) in Fig. 3a). This together with the above result of ^133^Cs MAS NMR indicates that CO_2_ adsorption occurs at Na^+^ cation sites on the surface of the 2D nanosheet. The dominant and additional low-field signals become intense for the Cs-type saponite (see (III) in Fig. 3a), providing the important information that the Cs-type saponite captures CO_2_ molecules more efficiently than that in the Na-type one. After heat treatment at 200 °C for 2 h, the dominant peak disappeared, whereas the broad hump at ~170 ppm remained (see (IV) in Fig. 2a), demonstrating that the intense and small signals are ascribed to physisorption (dominant) and chemisorption (secondary) on the nanosheet surfaces, respectively. It is reasonably inferred from the study of a ternary catalyst composed of copper, zinc oxide, and alumina^21^ that the broad signals at ~170 ppm originate from carbonate species, such as Cs_2_CO_3_.

It is of interest that both the physisorption and chemisorption for CO_2_ molecules occur at the alkali metal cations located on the surface of the 2D nanosheet. It is most probable that the CO_2_ physisorption on the nanosheet surface is caused by the quadrupole interaction between the alkali metal cations and CO_2_ molecules. Molecular orbital calculation, on the one hand, predicts the resultant carbonate species Cs_2_CO_3_ from CO_2_ chemisorption, in which the CO_2_ molecule stabilizes by bridging with two alkali cations on the nanosheet surface (Fig. 3b). The adsorption energy for the optimized structure calculated with the SCIGRESS program (Fujitsu Ltd. Japan) is −97.8 kcal/mol, which is similar to that of CO_2_ chemisorption onto the graphitic surface with two-bond conformation^22^. The concentrations of CO_2_ physisorption, *c*_PhysCO2_ and CO_2_ chemisorption, *c*_ChemiCO2_ for the Na- and Cs-type samples obtained from the total amount of CO_2_ and the ratio of two peak intensities in ^13^C MAS NMR spectra are listed in Table 1. It is noted that ~13% of the loaded CO_2_ is instantaneously chemisorbed as the carbonate species in both samples without employing strong acid solution at ambient pressure and temperature. As the concentration of Cs^+^ cations physisorbed on the surface of the 2D nanosheet is ~0.03 mmol/g (see Table 1), the cesium carbonate formed with two Cs^+^ cations on the nanosheet surface has the concentration of ~0.02 mmol/g. This is consistent with the concentration of CO_2_ chemisorption, *c*_ChemiCO2_ (see Table 1), signifying that Cs^+^ cations on the nanosheet surface dominantly take part in the CO_2_ chemisorption. On the other hand, the concentration of CO_2_ physisorption, *c*_PhysCO2_ is higher than that of Cs^+^ cations on the nanosheet surface. Presumably, a few CO_2_ molecules are physisorbed at Cs^+^ cations with the distance of several angstroms (see Fig. 3b).

## Discussion

According to a number of earlier works on zeolite materials with CO_2_ gas, the polarizing power of exchangeable alkali cations is one of the decisive factors for the capacity of CO_2_ gas adsorption^23^. The polarizing power of cations is inversely proportional to the ionic radius of alkali metal cations. The capacity of CO_2_ adsorption owing to the cation-quadrupole interaction thus increases as follows: Cs^+^<Rb^+^<K^+^<Na^+^<Li^+^^23^. This is in sharp contrast with the present observation that the concentration of both the physisorption and chemisorption increase for the Cs-type saponite. It is noted here that the fraction of open space with the size of ~9 Å for the Cs-type saponite probed by Ps is much higher than that of the Na-type one (see relative intensity *I*_2_ in Table 2). The open space formed by nanosheet overlap for the Cs-type saponite offers large enough surface area to accommodate CO_2_ molecules, thus being responsible for both the physisorption and chemisorption for CO_2_ molecules.

**Fig. 4:**
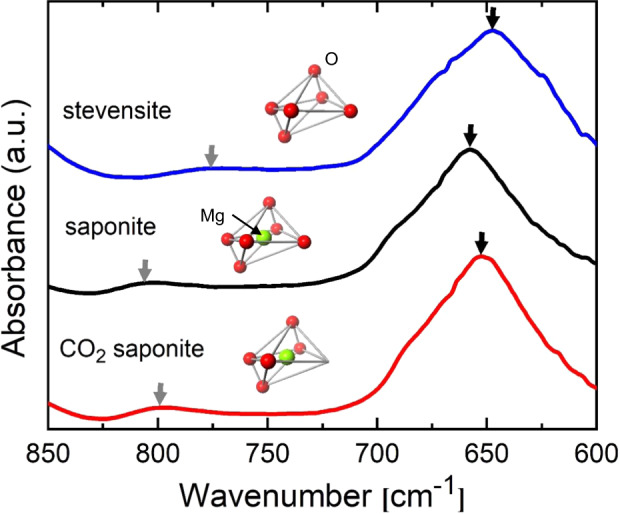
FT-IR spectroscopy. FT-IR spectra for CO_2_ unloaded (black curve) and loaded (red curve) saponite together with stevensite (blue curve). The absorption peaks of Si-O-Mg and Mg_3_OH bending vibrations are marked by black and gray thick arrows. The schematic illustrations of the disordered octahedra of stevensite, saponite, and CO_2_-loaded saponite are shown as the insets. Note that Mg and O sites are defective.

Here, we discuss why the chemisorption of CO_2_ gas molecules, i.e., ex situ mineral carbonation, instantaneously occurs in saponite samples without acid solution under the ambient condition. This unique adsorption nature is explained by the interior structure of the open nanospace characteristic for 2D materials. Saponite possess a 2:1 layered structure with 2D nanosheets consisting of tetrahedra and distorted octahedra. O and Si atoms are located at the vertices and central site of the tetrahedron, respectively. On the other hand, the O atoms or OH groups sit on the vertices of distorted octahedron, whereas a metallic Mg atom is located at the central site of octahedron (see the inset of Fig. 4). In the case of stevensite, the same family of saponite in aluminosilicate-type 2D materials, ca. 6.7% of the central Mg atoms in the octahedra are absent (see the inset of Fig. 4). An influence of Mg missing in the octahedron on the chemical bond is visible in the wavenumber region of 600–850 cm^−1^ in the FT-IR spectrum of stevensite. The FT-IR spectrum for the stevensite exhibits intense and small absorption peaks at the wavenumbers of around 650 and 775 cm^−1^, which can be ascribed to Si-O-Mg and Mg_3_OH bending vibrations, respectively^24^. The wavenumbers of the above bending vibration bands for the stevensite are lower than those of saponite, as the missing Mg atom causes lower frequencies in the both bending vibrations. It is noted here that the absorption peaks of Si-O-Mg and Mg_3_OH bending vibrations are red-shifted for CO_2_ loaded saponite as well implying atom missing in the octahedron (see Fig. 4). It is reasonably inferred that the above red-shift arise from the disappearance of O atoms from the vertices of the octahedra by the analogy of stevensite as illustrated in the inset of Fig. 4.

**Fig. 5:**
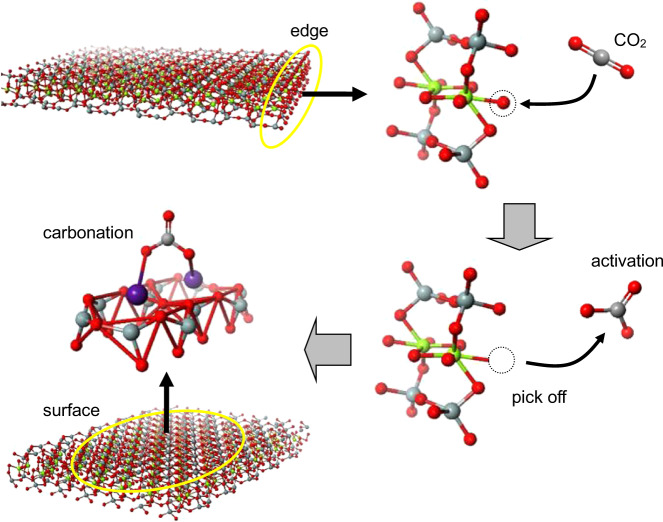
Mineral carbonation. Scenario of instantaneous ex situ mineral carbonation under ambient conditions. Red, gray, green, and purple atoms correspond to oxygen, carbon, magnesium, and cesium, respectively.

Based on the present findings of ^133^Cs and ^13^C MAS NMR and FT-IR spectroscopy, we can draw the scenario of instantaneous ex situ mineral carbonation as schematically illustrated in Fig. 5. In saponite, the O atoms are weakly bound to the octahedron by ionic bonding, in contrast to the strong semi-covalent bonding of O and Si atoms in the tetrahedron. CO_2_ gas molecules thus easily pick up the O atoms from the octahedra at the nanosheet edges in the interior of the above open spaces to be activated as the carbonate ions of CO_3_^2−^ type (see right-hand side of Fig. 5). The CO_3_^2−^ ions are then chemisorbed at the alkali metal cations on the surface of 2D nanosheets (see left-hand side of Fig. 5). The softness of the octahedra is also anticipated from the fact that the decomposition of octahedral sheets by mechanochemical milling proceeds prior to tetrahedral sheets^25^.

In the process of physical adsorption utilizing the physisorption of CO_2_ molecules, a solid adsorbent is exposed to the combustion gas containing acid gasses such as sulfur dioxide. The central concern in the research of solid-adsorbent development is hence always a lifetime of adsorbent material relevant to thermal/chemical stability and mechanical strength in addition to the production cost. Clay minerals, such as saponite, have high tolerances for acids and their cheapness should be emphasized more than anything, thus compensating for the shortcomings of current adsorbent materials mentioned above. In light of the fact that the peak of CO_2_ physisorption in the ^13^C MAS NMR spectrum completely disappeared upon heat treatment (see Fig. 3), the open space formed by nanosheet overlap could again offer large enough surface area to accommodate CO_2_ molecules. It is thus expected that the ability of CO_2_ physisorption reversibly occurs when the sample is heated under mild conditions as 200 °C. In addition to that, the ability of CO_2_ chemisorption, i.e., ex situ mineral carbonation, instantaneously emerging without chemical solution under the ambient condition is highly beneficial over amine-based systems with respect to less-energy intensiveness, cost effectiveness, and environmental friendliness. As the nuclear accident in Fukushima, the large quantities of Cs-contaminated soil containing clays generated from decontamination activities have been stored throughout prefecture. The reusing and recycling of contaminated soil on the basis of criteria for both environmental and human impacts upon proper optimization is thus awaited in the near future. The Ministry of the Environment of Japan has promoted the recycling of decontamination soil with a radioactivity level below 8000 Bq/kg for specific construction purposes as, e.g., construction of road^26^. We believe that the present findings on the ability of CO_2_ adsorption in well-known clays could open up the recycling strategy of low contaminated soil containing clays with respect to the environment-friendly construction materials.

## Methods

### Materials

Synthetic Na-type saponite Na_0.66_[Mg_5.34_Li_0.66_]Si_8_O_20_[OH]_4_ produced by Kunimine Industries Co. Ltd., Japan, was employed in the present work. Cs loading was conducted by impregnating the Na-type saponite with a 1 M CsCl solution for ion exchanging with Cs. Aqueous solution was completely eliminated from the sample, which is referred to as Cs-type saponite. The chemical element in the Cs-type sample was examined by ICP spectroscopy (ICPS-8100, Shimadu). The sample was first treated at 423 K for 8 h under a vacuum condition of ~10^–5^ Torr in order to eliminate physisorbed H_2_O molecules. The dehydrated sample was successively replaced with 1-mbar CO_2_ atmosphere for CO_2_ loading without any contact to air and then kept there for 30 min, which was then analyzed for CHN using an elemental analyzer and ^133^Cs MAS NMR. In parallel to that, the dehydrated sample was exposed to a ^13^C-enriched CO_2_ (^13^CO_2_) atmosphere at 50-mbar for 30 min, which was subjected to ^13^C MAS NMR.

### Positronium lifetime spectroscopy

The sizes of open nanospaces and their fractions were investigated by Ps annihilation lifetime spectroscopy employing digital oscilloscope (WaveSurfer 10, Teledyne LeCroy). The positron source (^22^Na), sealed in a thin foil of Kapton, was mounted in a sample-source-sample sandwich configuration. The 1.27 MeV positron birth γ ray from a ^22^Na source and one of the 511 keV γ rays emitted as a result of positron annihilation in the samples are detected by BaF_2_ scintillators with 1'' diameter × 1'' thickness coupled with photomultiplier tubes (H3378-51, HAMAMATSU). Positron lifetime spectra were numerically analyzed. A fraction of energetic positrons injected into samples forms the bound state with an electron, Ps. Singlet *para*-Ps (*p*-Ps) with the spins of the positron and electron antiparallel and triplet *ortho*-Ps (*o*-Ps) with parallel spins are formed at a ratio of 1:3. Hence, three states of positrons: *p*-Ps, *o*-Ps, and free positrons exist in samples. The annihilation of *p*-Ps results in the emission of two γ-ray photons of 511 keV with a lifetime ~125 ps. Free positrons are trapped by negatively charged parts, such as polar elements, and annihilated into two photons with a lifetime ~450 ps. The positron in *o*-Ps undergoes two-photon annihilation with one of the bound electrons with a lifetime of a few ns after localization in nanospaces. The last process is known as *o*-Ps pick-off annihilation and provides information on the free volume size *R* through its lifetime *τ*_*o*–Ps_ based on the Tao-Eldrup model^27,28^.

1 $$\tau _{o - {\rm{Ps}}} = 0.5\left[ {1 - \frac{R}{{R_0}} + \frac{1}{{2\pi }}{\mathrm{sin}}\left( {\frac{{2\pi R}}{{R_0}}} \right)} \right]^{ - 1}$$

where *R*_0_ = *R* + ∆*R*, and ∆*R* = 0.166 nm is the thickness of homogeneous electron layer in which the positron in *o*-Ps annihilates. On the one hand, the relative intensity of *τ*_*o*–Ps_ is assumed to be correlated with the amount of open nanospaces.

### Solid-state nuclear magnetic resonance

The ^133^Cs and ^13^C MAS NMR experiments were performed using a Bruker Avance III 400WB spectrometer at the resonance frequencies of 52.5 MHz and 100.6 MHz, respectively. ^133^Cs MAS NMR spectra were recorded with single-pulse excitation of 2.0 µs and the repetition time of 3 s. A sample spinning rate of 22 kHz was used and 24,000 scans were accumulated. The chemical shifts were referenced to a 1.0 M solution of CsCl. ^13^C MAS NMR measurements were performed via exciting the ^13^C spins with single-pulses of 2.0 µs and with a repetition time of 20 s thus avoiding relaxation effects by *T*_1_. The sample spinning rate was 8 kHz and 320 scans were collected for each spectrum. The chemical shifts of the ^13^C nuclei in the adsorbed organic species were determined with respect to tetramethylsilane as the external reference with an accuracy ±1 ppm.

### FT-IR spectroscopy

Attenuated total reflection (ATR) FT-IR spectra were measured using a Nicolet iS5 FT-IR spectrometer (Thermo Fisher Scientific Inc.) using the ATR device with a diamond crystal plate. All the FT-IR spectra were measured at room temperature with the resolution of 4 cm^−1^. The measurements were repeated 100 times and the final spectra were obtained by averaging them. OMNIC 8.2 software was used to display absorbance spectra by converting ATR data, where the absorption band in the range of wavenumbers from 600 to 850 cm^−1^ is focused.

## Supplementary information

Peer Review File

## Data Availability

The data that support the findings of this study are available from the corresponding author upon reasonable request.
